# Central motor conduction time in spinocerebellar ataxia: a meta-analysis

**DOI:** 10.18632/aging.104181

**Published:** 2020-11-20

**Authors:** Zhi-Chao Tang, Zhao Chen, Yu-Ting Shi, Lin-Lin Wan, Ming-Jie Liu, Xuan Hou, Chun-Rong Wang, Hui-Rong Peng, Lin-Liu Peng, Rong Qiu, Bei-Sha Tang, Hong Jiang

**Affiliations:** 1 Department of Neurology, Xiangya Hospital, Central South University, Changsha, Hunan, China; 2 Key Laboratory of Hunan Province in Neurodegenerative Disorders, Central South University, Changsha, Hunan, China; 3 National Clinical Research Center for Geriatric Disorders, Xiangya Hospital, Central South University, Changsha, Hunan, China; 4 Laboratory of Medical Genetics, Central South University, Changsha, China; 5 Department of Pathology, Xiangya Hospital, Central South University, Changsha, Hunan, China; 6 School of Computer Science and Engineering, Central South University, Changsha, Hunan, China

**Keywords:** spinocerebellar ataxia, subtypes, transcranial magnetic stimulation, central motor conduction time, meta-analysis

## Abstract

The dominantly inherited spinocerebellar ataxias (SCAs) are a large class of neurodegenerative diseases. Transcranial magnetic stimulation has been used to evaluate the function of the pyramidal tract, and central motor conduction time (CMCT) is one index used to detect pyramidal tract dysfunction. We conducted a comprehensive search of PubMed, Embase and Web of Science. Eight eligible studies were included in the meta-analysis. For upper limb CMCT, the mean difference (95% confidence interval (CI)) between the combined SCA group and the control group was 2.24 [1.76-2.72], while the mean differences (95% CIs) between the subtypes and the control group were as follows: 4.43 [3.58-5.28] for SCA1, 0.25 [-0.15,0.65] for SCA2, 1.04 [-0.37,2.46] for SCA3 and 0.49 [-0.29,1.28] for SCA6. Additionally, SCA1 significantly differed from SCA2 and SCA3 in terms of CMCT (P=0.0006 and P=0.010, respectively). We also compared lower limb CMCT between the SCA2 and control groups. The mean difference (95% CI) was 6.58 [4.49-8.67], which was clearly statistically significant. The differences in CMCT values among different subtypes suggests diverse pathological mechanisms. In general, CMCT is a promising objective index to judge the severity of disease deserving further investigation.

## INTRODUCTION

The dominantly inherited spinocerebellar ataxias (SCAs) are a large class of neurodegenerative diseases [1]. More than 40 genetically distinct SCAs have been defined, of which the most common (SCA1, SCA2, SCA3, SCA6 and SCA7) are caused by amplification of the CAG repeat encoding glutamine in the affected genes [2]. Considerable variability has been found in the disease characteristics of SCAs, partly because of different degrees of CAG repeat amplification. More repeat expansion is always associated with earlier symptom onset and broader neurological symptoms. Additionally, the neuropathological and clinical manifestations of each subtype are not exactly the same [3]. SCA1 can emerge at any developmental stage from infancy to adulthood. Among the SCA subtypes, SCA1 develops the fastest and is accompanied by cerebellar and noncerebellar symptoms. SCA2 usually manifests as progressive ataxia, oculomotor retardation and sensorimotor neuropathy. Extrapyramidal manifestations, including Parkinson’s disease, motor weakness, ocular palsy and cognitive impairment, may also occur. SCA3, also known as Machado-Joseph disease, is characterized by progressive ataxia and spasm [4–6]. SCA6 starts later than other polyQ ataxias; this subtype is considered a relatively pure cerebellar syndrome with few extracerebellar signs [7]. Although the main symptom of SCAs is ataxia caused by cerebellar damage, patients with SCAs also frequently have pyramidal symptoms, which are often aggravated as the disease progresses. However, there are few quantitative studies on the extent of damage to the pyramidal tract in SCA patients.

Transcranial magnetic stimulation (TMS) is a relatively safe and noninvasive means of electrically stimulating the brain by electromagnetic induction. This technique is currently being used in the diagnosis and treatment of many diseases. Because of its convenience and noninvasiveness, TMS has a high rate of patient compliance [8, 9]. TMS has been used to evaluate the function of the pyramidal tract, and central motor conduction time (CMCT) is one of the indices calculated from motor evoked potentials (MEPs) to measure pyramidal tract dysfunction. As a marker of conduction deceleration caused by abnormal upper motor neurons, CMCT has been explored in several diseases, such as cerebral small vessel disease and hereditary spastic paraplegia [10]. However, patients with SCAs often suffer from pyramidal tract involvement, and different subtypes do not have exactly the same pathogenesis. In this context, can CMCT be used as a marker of SCA pathogenesis and a means of differentiating SCA subtypes?

Studies that address this question are scarce. In some previous studies, TMS was used in patients with SCAs, and CMCT showed different degrees of prolongation in patients compared with the control group [16]. However, the sample sizes of these studies were small, and often, only one subtype of patients was selected. The aim of the present meta-analysis was to integrate the data, explore the comprehensive performance of CMCT in SCAs, identify the differences in CMCT among different subtypes and reveal the pathological differences among different subtypes of SCAs. We also investigated CMCT as a potential marker for observing the progress and prognosis of the disease, thus providing a future direction for the diagnosis and treatment of SCAs [14, 15].

## RESULTS

### Search results

**Figure 1:**
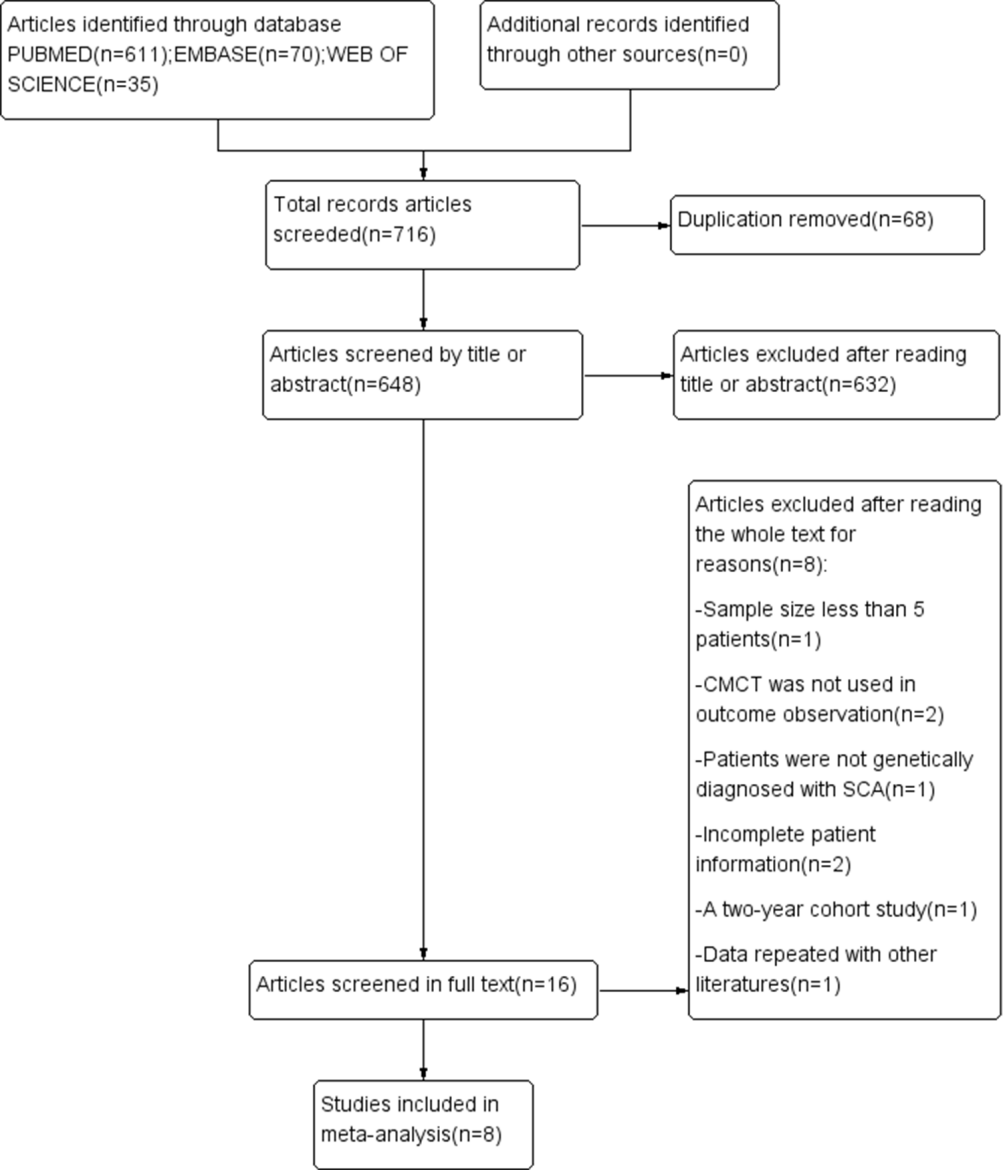
**PRISMA flowchart of the studies included in the meta-analysis.** SCA, spinocerebellar ataxia; CMCT, central motor conduction time.

By using keywords in the PubMed, Embase and Web of Science databases, we obtained 648 records after removing duplicate records. After the preliminary reading of the title and abstract, 16 articles that fit the topic were read in full-text form. Based on the exclusion criteria, 8 articles were eliminated (see Figure 1 for the reasons), and 8 articles were included in the meta-analysis.

### Study characteristics

The characteristics of the meta-analysis studies are shown in Table 1. In general, eight studies involved 4 common subtypes of SCAs. The studies involved different regions and countries over a long time span (from 1998 to 2016). By combining data across studies, we obtained new SCA samples (SCA1 = 28, SCA2 = 81, SCA3 = 39 and SCA6 = 25). The total number of controls was 200. From the observation data, we found that among the four subtypes included in this study, SCA6 had the latest onset age and the longest course of disease. There was no significant difference among the other three subtypes. Regarding CAG length, SCA6 tended to be the shortest, and SCA3 tended to be the longest, while SCA1 and SCA2 were not significantly different [16–23].

**Table 1:** Study and patient characteristics.

**Genotype**	**Author**	**Year**	**Country/district**	**Sample size**	**Men (n)**	**Age (y)**	**Disease duration (y)**	**CAG**	**Location**	**CMCT (ms)**
SCA1	T Yokota	1998	Japan	10	NR	41.2±15.1	7.1±5.2	NR	upper limbs/lower limbs	9.5±1.6/21.3±3.5
	Peter Schwenkreis	2002	Germany	3	3	42.3±8.7	5.7±5.9	50.7±4.6	upper limbs	10.3±4.7
	Ketan Jhunjhunwala	2013	India	15	11	28.0±10.9	4.9±3.1	55.6±7.3	upper limbs	8.9±2.6
SCA2	T Yokota	1998	Japan	8	NR	46.8±10.0	14.3±5.7	NR	upper limbs/lower limbs	5.1±0.4/13.1±1.6
	D.A. Restivo	2000	Italy	18	12	48.4±14.6	12.3±7.2	40.6±2.9	upper limbs/lower limbs	6.3±1.4/19.2±7.0
	Peter Schwenkreis	2002	Germany	7	3	36.6±4.1	8.7±3.5	40.5±1.7	upper limbs	6.6±0.7
	Ketan Jhunjhunwala	2013	India	11	10	29.9±9.5	6.0±5.6	43.1±2.0	upper limbs	6.5±1.1
	Luis Velázquez-Pérez	2016	Cuba	37	11	40.4±11.0	presymptomatic	36.3±2.3	lower limbs	20.2±7.9
SCA3	T Yokota	1998	Japan	10	NR	43.9±11.0	8.2±5.2	NR	upper limbs/lower limbs	4.5±0.8/13.2±0.9
	Peter Schwenkreis	2002	Germany	12	8	44.8±12.8	8.5±4.6	73.0±3.6	upper limbs	6.9±0.9
	Ketan Jhunjhunwala	2013	India	6	2	38.8±5.5	6.0±2.5	71.3±1.5	upper limbs	6.8±1.5
	Michelle A. Farrar	2016	Australia	11	5	44.9±11.6	9.8±6.1	70.9±3.0	upper limbs	7.5±0.4
SCA6	Peter Schwenkreis	2002	Germany	9	6	56.1±10.0	6.9±4.0	23.1±2.1	upper limbs	6.9±1.1
	Jen-Tse Chen	2004	Taiwan	9	5	55.7±8.6	11.1±6.8	NR	upper limbs/lower limbs	9.1±1.3/18.1±1.9
	Kenji Sakuma	2005	Japan	7	1	68.4±7.0	21.1±10.6	24.0±2.4	upper limbs	7.1±1.7
Control	T Yokota	1998	Japan	16	NR	NR	-	-	upper limbs/lower limbs	4.8±1.1/13.0±1.7
	D.A. Restivo	2000	Italy	20	12	44.4±14.8	-	-	upper limbs/lower limbs	5.9±0.8/12.1±3.1
	Peter Schwenkreis	2002	Germany	14	9	40.1±13.3	-	-	upper limbs	6.6±1.1
	Jen-Tse Chen	2004	Taiwan	10	6	52.1±5.0	-	-	upper limbs/lower limbs	6.9±0.5/15.0±1.0
	Kenji Sakuma	2005	Japan	9	3	66.0±15.8	-	-	upper limbs	6.1±1.2
	Ketan Jhunjhunwala	2013	India	32	19	29.5±4.4	-	-	upper limbs	4.8±0.6
	Michelle A. Farrar	2016	Australia	62	31	45.8±NR	-	-	upper limbs	5.3±0.2
	Luis Velázquez-Pérez	2016	Cuba	37	11	40.4±11.25	-	-	upper limbs/lower limbs	13.9±1.7

Age, disease duration, CAG and CMCT are summarized as mean±SD. SCA, spinocerebellar ataxia; CAG, length of expanded repeats allele; CMCT, central motor conduction time; NR, not reported.

Because most data described the CMCT in the upper limbs, they were used for the analysis and comparison of different SCA subtypes. CMCT data of the lower limbs were discussed only in the comparison of SCA2 with the control group.

### CMCT comparison in upper limbs

**Figure 2:**
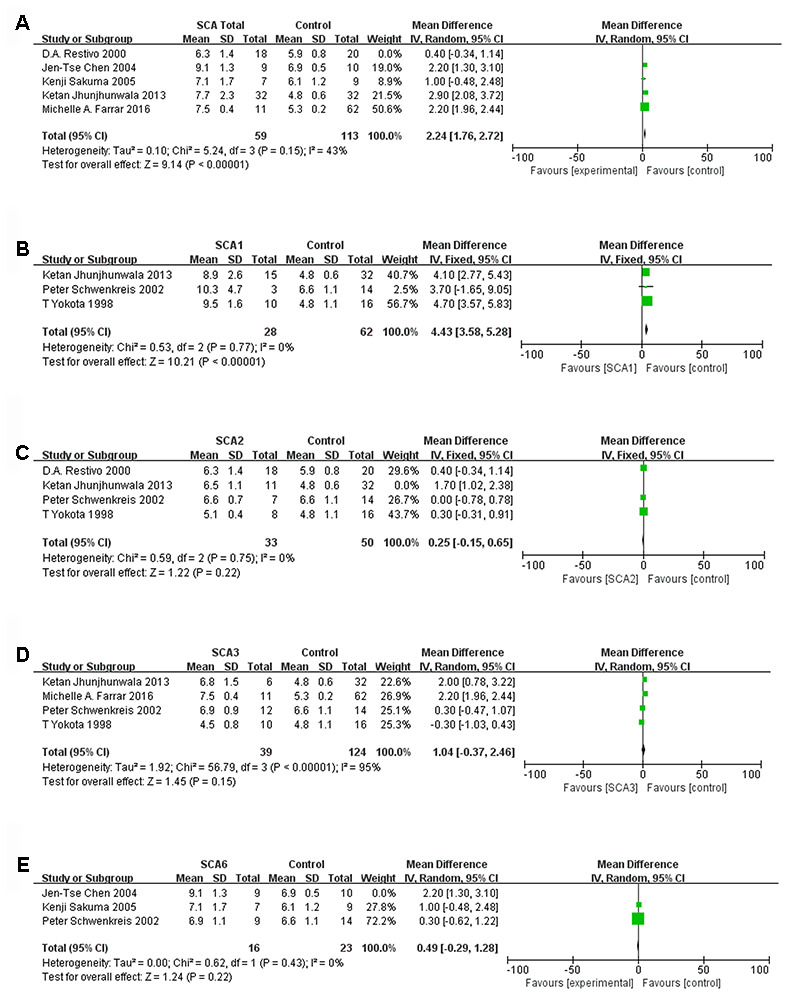
****Forest plot of (**A**) SCA total, (**B**) SCA1, (**C**) SCA2, (**D**) SCA3, (**E**) SCA6 after sensitivity analysis.

Five of the eight articles were selected for comparisons between the overall SCA group and the control group. Three articles were excluded because they did not include the total standard deviation or because they used lower limb CMCT. The results are shown in forest plots (Figure 2A). The total sample size of the SCA group was 77, and that of the control group was 133. There were statistically significant differences between the SCA and control groups (d, 1.79; 95% CI, 0.97 to 2.62; P < 0.0001). However, the I² value of 85% indicated that the heterogeneity was high. After the study by D.A. Restivo (2000) was excluded, the sensitivity analysis found that the I² was reduced to 43%. The sample sizes of the SCA and control groups changed to 59 and 113, respectively, and statistically significant differences still existed (d, 2.24; 95% CI, 1.76 to 2.72; P < 0.00001).

**Figure 3:**
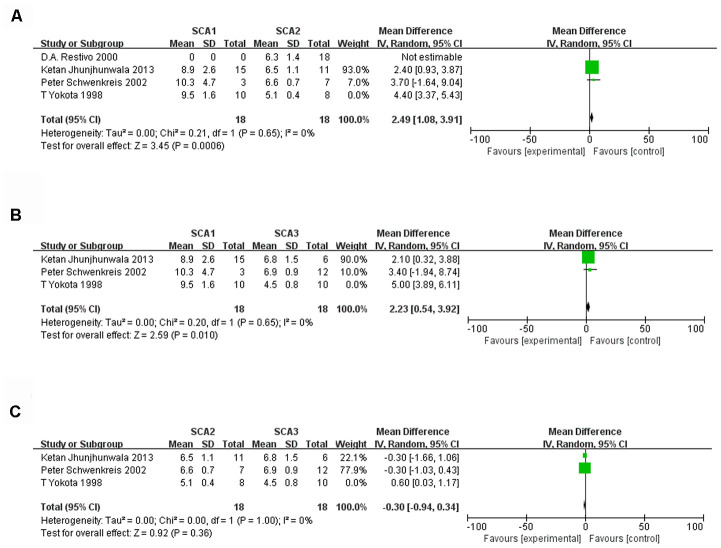
****Forest plot of the comparison between SCA1 and SCA2 (**A**), SCA1 and SCA3 (**B**), SCA2 and SCA3 (**C**) after sensitivity analysis.

When each subtype of SCA was analyzed separately, the heterogeneity of the three studies involving SCA1 was low, with I² = 0%. The sample size was 28:62 (SCA1:control), and significant differences were observed (d, 4.43; 95% CI, 3.58 to 5.28; P < 0.00001, Figure 2B). When we removed the article by Ketan Jhunjhunwala (2013), the 3 remaining studies of SCA2 had a sample size of 33:50 (SCA2:control) and low heterogeneity (I² = 0%). However, the mean difference (95% CI) was 0.25 (-0.15, 0.65), which was not statistically significant (Figure 2C). A sensitivity analysis of four SCA3 studies showed that heterogeneity was high, and the results were not statistically significant (Figure 2D). After the paper by Jen-Tse Chen (2004) was excluded, the heterogeneity of the SCA6 studies was very low (Figure 2E). Nevertheless, there was no significant difference between the SCA6 group and the control group (P=0.22). Data from three studies were included in the comparative analysis of the SCA1, 2 and 3 subtypes. We found that there was significant heterogeneity (I^2^ was more than 50%), but after we excluded the study by T Yokota (1998), the heterogeneity of three subtype comparisons became extremely low (I^2^ = 0%). We observed that the sample sizes for SCA1, SCA2 and SCA3 were all 18 (Figure 3A–3C). Only the comparisons of SCA1 with SCA2 and SCA3 yielded statistically significant results (P=0.0006 and P=0.010, respectively).

### CMCT comparison for lower limbs

**Figure 4:**
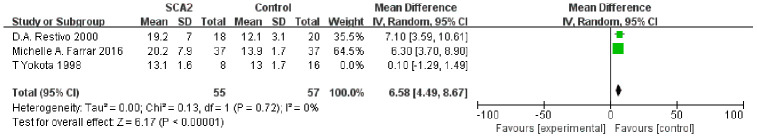
**Forest plot of SCA2 and control group lower limb values after sensitivity analysis.**

Because there were very few data on lower limb CMCT, we analyzed only the data from the SCA2 and control groups. The results are displayed in Figure 4. To reduce heterogeneity, we removed the study by T Yokota (1998) in the sensitivity analysis, and I^2^ became 0%. The sample size was 55:57 (SCA2:control), and the difference was statistically significant (d, 6.58; 95% CI, 4.49 to 8.67; P < 0.00001).

## DISCUSSION

This study is the first meta-analysis of CMCT in patients with SCAs. Compared with the control group, the SCA showed a trend toward prolonged CMCT of the upper limb, although there was some heterogeneity. To analyze and reduce the heterogeneity, we organized the analysis into groups according to SCA subtype. Among the various subtypes, the difference was most obvious for SCA1. In the SCA2, SCA3, and SCA6 groups, the CMCT of the upper limbs was not significantly different from that of the control group. The comparison among subtypes showed that SCA1 was significantly different from SCA2 and SCA3. For the lower limbs, patients with SCA2 showed significantly prolonged CMCT compared to the control group.

Sensitivity analysis revealed that the three articles on SCA1 had good homogeneity, while the four articles on SCA3 had high heterogeneity. The reasons for the high heterogeneity in SCA3 might be as follows. First, the sample size was quite small. Second, Table 1 shows that in the study by Ketan Jhunjhunwala (2013) [20], the study population was younger and the duration of disease was relatively short. Additionally, in the study by T Yokota (1998), the ratio of males to females and the CAG repeat number were not reported. Third, there were differences in the experimental methods. In the study by Ketan Jhunjhunwala (2013), the inner diameter of the coil was 70 mm instead of the 90 mm used in other studies. It was evident that the study by Ketan Jhunjhunwala (2013) [20] contributed greatly to the heterogeneity of the SCA2 analysis. At the same time, the study by Jen-Tse Chen (2004) [22] significantly contributed to the heterogeneity of the SCA6 studies.

The reasons for the heterogeneity introduced by those two studies are as follows. First, there were differences in the characteristics of the study populations and the control groups. For example, in the study of SCA2 by Ketan Jhunjhunwala (2013) [20], the disease duration of the patients was relatively short, and the patients and controls were relatively young. In the study of SCA6 by Jen-Tse Chen (2004) [22], the patients and the control group came from Asia, and the specific CAG repeat copy numbers were not directly given, which may represent differences from other studies. Furthermore, although CMCT was calculated by the F-wave method, the parameter settings for TMS (such as the width and angle of the coil) were not identical, and the recording methods were different, both of which could have resulted in heterogeneity. Remarkably, T Yokota (1998) [16] contributed to the heterogeneity of three SCA subtype analyses, particularly the lower limb CMCT analysis of SCA2. This was probably because the study was considerably divergent from the others and used different parameters. Experimental artifacts could also exist.

SCA1, 2 and 3 are autosomal dominant disorders characterized by cerebellar ataxia; these disorders manifest as oculomotor nerve abnormalities, pyramidal or extrapyramidal features and peripheral neuropathy. CMCT is a quantitative neurophysiological measurement method for objectively evaluating corticospinal tract conduction. This measurement reflects the conduction between the primary motor cortex and spinal cord. There may be several mechanisms of CMCT prolongation. First, axons or synapses in the pyramidal tract may gradually decrease in number over the course of the disease. In addition, axons of the pyramidal tract may become demyelinated or atrophied. Moreover, decreased excitability of the α-motor neurons may prolong the time it takes for the membrane potential of those neurons to reach the threshold for an action potential [16]. Previous autopsies of SCA1 patients showed selective loss of large fibers or axon atrophy and demyelination of the corticospinal tract, which could explain the significant prolongation of CMCT in SCA1. However, some previous studies have also shown significant CMCT prolongation in SCA2 and SCA3. Extensive cortical degeneration has also been observed in some SCA2 autopsies [17]. The dysfunction of pyramidal cells in patients with SCA3 (e.g., exaggerated stretch reflexes) also suggests the involvement of the motor cortex. This was not consistent with our results, probably because the sample size was insufficient or because patients were in different stages of the disease. The heterogeneity of the studies also accounted for the result. We discovered that there was a significant difference in lower limb CMCTs between the SCA2 patients and the control group, probably because of the differences in stimulation and recording sites; this difference indicates that CMCT is more sensitive in the lower limbs than in the upper limbs [22]. As SCA6 is a “pure cerebellar” subtype, we were not surprised by the results. Although extracerebellar symptoms are also occasionally reported in SCA6 [20], we believe that cerebellar lesions, which would not cause significant changes in CMCT, are still the main mechanism of SCA6.

In general, compared with the control group, CMCT of SCA1 and SCA2 types was significantly prolonged, while there was no significant difference in SCA3 or SCA6 types. Our meta-analysis provides a new direction for future studies on the pathophysiology of SCAs. First, the difference in CMCT subtypes could reflect the involvement of the motor cortex and conduction pathway, thus providing clinical evidence for the pathology of the disease. Second, there were obvious differences in CMCT among the SCA1, SCA2, SCA3 and control groups, suggesting that CMCT could be used as an auxiliary means of disease classification. Additionally, as a noninvasive examination, it could be used as a potential follow-up evaluation index alongside a clinical scale in cohort studies to estimate the progression of the disease and the effect of therapy.

We have several suggestions for future research. Above all, it would be best to expand the sample size and ensure a lack of confounders between patients and controls. Furthermore, TMS methods and parameters should be standardized to facilitate the sorting and analysis of the results. Both upper limb and lower limb CMCT should be recorded. Moreover, we predicted that the upper motor neurons of SCA2 and SCA3 patients might also be damaged; therefore, we hope that CMCT studies of SCA2 and SCA3 with large samples will be carried out in the future. Finally, CMCT combined with clinical follow-up could be carried out in patients with SCA, especially those with SCA1. The sensitivity and stability of CMCT as an indicator of disease progression could be observed. Our research also had some limitations. For example, some studies were quite old. Additionally, there was heterogeneity among different studies (regarding the study populations, recording methods, etc.), and the number of studies in subgroup analyses was low after the sources of heterogeneity were excluded. Nevertheless, we did include a larger sample than any previous study, and we were able to derive a meaningful conclusion. We hope that subsequent studies will further expand the data.

## CONCLUSIONS

CMCT prolongation has been observed in different subtypes of SCAs. The difference in CMCT values among different subtypes suggests diverse pathological mechanisms. Our study opens the possibility of performing quantitative analysis and dynamic observation of the extent of damage to the pyramidal tract in SCA patients. As an objective index with which to determine the severity of the disease, CMCT has broad prospects for application, although they need to be confirmed in the future.

## MATERIALS AND METHODS

We conducted this meta-analysis based on the Meta-analysis Of Observational Studies in Epidemiology (MOOSE) guidelines [24] and the corresponding MOOSE checklist [11].

### Literature search strategies

To comprehensively examine the relationship between SCA and CMCT, we conducted a comprehensive search of PubMed and Web of Science using the following keywords: [Mesh] “Spinocerebellar Ataxia” or “spinocerebellar ataxia” or “spinocerebellar ataxias” or “spinocerebellar degeneration” or “spinocerebellar atrophy” or “spinocerebellar degenerations” or “spinocerebellar atrophy” or “dominant ataxia” or “hereditary ataxia” or “SCA” or “olivopontocerebellar atrophy” or “Wadia-Swami Syndrome” or “Machado-Joseph disease” in combination with [Mesh] “Transcranial Magnetic Stimulation” or “transcranial magnetic stimulation” or “TMS” or “central motor conduction” or “central motor conduction time”. For Embase, the retrieval strategy used was as follows: (‘spinocerebellar’/exp OR spinocerebellar) AND (‘ataxia’/exp OR ataxia) AND (‘transcranial’/exp OR transcranial) AND (‘magnetic’/exp OR magnetic) AND (‘stimulation’/exp OR stimulation). We limited the search to human studies published in English before April 2020. The references of the articles were also comprehensively searched to identify articles that were missed in the database search, and these articles were judged according to the same selection criteria as the search results.

### Study selection and data extraction

We applied several inclusion criteria. First, the study needed to involve SCA patients receiving TMS. The parameters of this group were noted. Second, patient information needed to be complete or nearly complete. Third, an age-matched control group was required, and the parameters of the control group needed to be specified.

Studies were excluded if any of the following criteria were met: the sample size was too small (n<5), or the patient information was inadequate; the patients were not diagnosed with SCA by molecular techniques [8]; TMS was applied in repeated sessions rather than a single session; CMCT values were not directly provided in the article; or the method used to calculate CMCT was not the F-wave method [11].

Data were extracted by two independent reviewers (Z.C.T., M.J.L.), and if there was any disagreement, the data were sent to a third reviewer (L.L.W.) for further exploration. The extracted information was as follows: names of authors, publication year, country, ataxia genotype, sample size, population features (age, sex, disease duration, number of CAG repeats), measurement location (upper limbs/lower limbs) and CMCT.

### Quality and risk of bias assessment

To evaluate the risk of bias, an independent analysis of all eligible studies was conducted using the modified Newcastle-Ottawa Scale assessment checklist [25], which was designed to assess three main areas of potential bias in cohort studies: the selection of study groups, the comparability of these groups, and the ascertainment of outcomes. We deleted the section that involved longitudinal studies, thus reducing the maximum possible score on the checklist to 6. All eight studies included in the meta-analysis scored 5 or 6, which indicated a low risk of bias (Supplementary Table 1). We also analyzed the publication bias of the overall SCA research data. They were evenly distributed on both sides in the funnel plot (Supplementary Figure 1).

### Statistical analysis

The characteristics of each study are summarized in Table 1, and quantitative variables are expressed as the mean and SD. Because the CMCT measurement sites considered in the study included both the upper limbs and lower limbs, we analyzed the data according to the body part measured. Additionally, different studies focused on different SCA subtypes. To explore the differences in CMCT among the SCA subtypes, a statistical analysis of each SCA subtype was carried out after the aggregate analysis of SCAs as a whole.

Forest plots were used for the statistical analysis of the data. Differences between two groups are reported as the mean difference (95% confidence interval). For all tests, P < 0.05 was deemed to be significant. The I² statistic measures the percentage of total variation introduced by heterogeneity rather than by chance. This statistic was used to quantify heterogeneity, with 25%, 50% and 75% indicating low, medium and high degrees of heterogeneity [12], respectively. For results with medium or higher heterogeneity (I² is greater than 50%), we used a random-effects model to explain the possible heterogeneity among the studies. We also performed a sensitivity analysis to reduce heterogeneity [13]. All statistical analyses were performed using RevMan statistical software (version 5.3).

## Supplementary Material

Supplementary Figure 1

Supplementary Table 1
