# Supplementary figures and images for: Central motor conduction time in spinocerebellar ataxia: a meta-analysis
Source: Aging (Albany NY). 2020 Nov 20;12(24):25718–29. doi: 10.18632/aging.104181 (PMC7803510; doi:10.18632/aging.104181)

SUPPLEMENTARY FIGURE

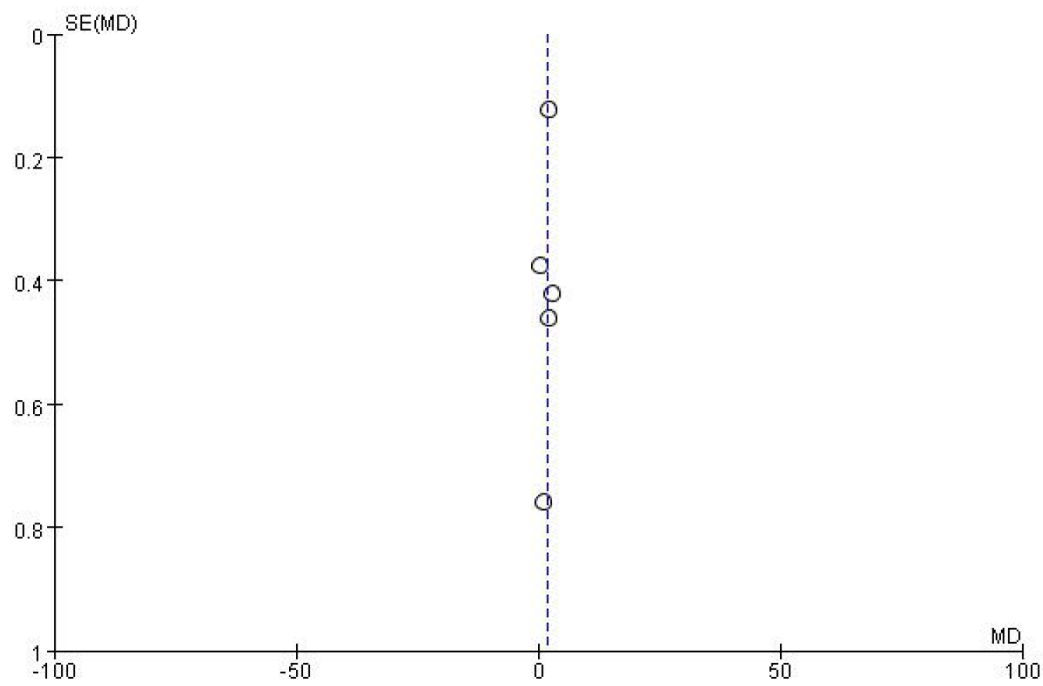

Supplementary Figure 1. Funnel plot for studies of SCA total.

Supplement: Supplementary Figure 1 [file aging-12-104181-s001.pdf]
