# Supplementary material for: Central motor conduction time in spinocerebellar ataxia: a meta-analysis
Source: Aging (Albany NY). 2020 Nov 20;12(24):25718–29. doi: 10.18632/aging.104181 (PMC7803510; doi:10.18632/aging.104181)
Supplement: Supplementary Table 1 [file aging-12-104181-s002.pdf]

SUPPLEMENTARY TABLE

Supplementary Table 1. Modified Newcastle-Ottawa Quality Assessment Scale.

Study	Selection	Comparability	Outcome	Total number of stars
T Yokota(1998)	★★★	★	★	5
Peter Schwenkreis(2002)	★★★	★★	★	6
Kenji Sakuma(2005)	★★★	★	★	5
D.A. Restivo(2000)	★★★	★★	★	6
Ketan Jhunjhunwala(2013)	★★★	★	★	5
Michelle A. Farrar(2016)	★★★	★★	★	6
Jen-Tse Chen(2004)	★★★	★★	★	6
Luis Velázquez-Pérez(2016)	★★★	★★	★	6

A maximum of 6 stars can be granted in total. Selection=maximum of 3 stars. Comparability = maximum of 2 stars. Outcome = maximum of 1 star.
